# Reproduction and population structure of the sea urchin *Heliocidaris crassispina* in its newly extended range: The Oga Peninsula in the Sea of Japan, northeastern Japan

**DOI:** 10.1371/journal.pone.0209858

**Published:** 2019-01-02

**Authors:** Wenping Feng, Nobuyasu Nakabayashi, Kazumi Narita, Eri Inomata, Masakazu N. Aoki, Yukio Agatsuma

**Affiliations:** 1 Graduate School of Agricultural Science, Tohoku University, Aramaki, Aoba, Sendai, Miyagi, Japan; 2 Akita Prefectural Institute of Fisheries, Oga, Akita, Japan; Shanghai Ocean University, CHINA

## Abstract

Ocean warming has facilitated the range expansion of commercially important sea urchin species to higher latitudes. *Heliocidaris crassispina* was recorded to extend northward to Toga Bay along the Oga Peninsula, Japan following an increase in seawater temperatures, and replacement of local sea urchin species *Mesocentrotus nudus*. In order to identify evidence of adaptation occurring in response to a range extension of *H*. *crassispina* to the newly extended environments, we randomly collected 106 *H*. *crassispina* in August 2014 in Toga Bay, determined the growth and age composition and examined gonad traits (size, color and development). To confirm the gonad development, 30 *H*. *crassispina* with > 30 mm diameter were collected in July, August and September 2017. We found slower growth in the extended range than the central range. More delayed gonad development of males than those of females and a large variety of developmental stages in the acini of testis indicated that the spawning of both sexes of the sea urchins were asynchronous. In terms of gonad color, L* (lightness) values increased with increasing GI, while b* (yellowness) values decreased with increasing age. The population consisted of seven year-classes from 2006 to 2012, suggesting persistent juvenile recruitment. Long-term water temperature data indicated that the range extension of *H*. *crassispina* was due to ocean warming, in particular during the summer spawning season.

## Introduction

Climate change mainly presented by global warming has caused increase in seawater temperature [1–2]. It potentially propels extension of marine organisms to high latitude areas [3]. Sea urchin is one of the apparent and ecologically important species due to its ability to overgraze seaweed beds and create “barren” habitats [4–5]. Seawater temperature is an important factor for gametogenesis [6–7], spawning [8–9] and larval development [10–12]. High water temperatures during the larval period of the sea *Hemicentrotus pulcherrimus* from 1989 to 1991 were coincide with an episodic recruitment event that extended the range of this species in the Sea of Japan from south to northern Hokkaido, Japan [13], where their fast growth rate and a strong gametogenic cycle with high gonad production were identified [14].

The diadematid sea urchin *Centrostephanus rodgersii* extended its range to the eastern Tasmania due to increased larval dispersal [15–16] driven by the poleward advance of the East Australian Current by ~ 350 km over the past 60 years [17]. In eastern Tasmania, *Centrostephanus rodgersii* displayed a strong seasonal cycle in gonad production with a major spawning occurring in winter (~ August) at minimum annual water temperature and produced viable gametes [18]. *Centrostephanus rodgersii*, which has also expanded its range to northern New Zealand in the last 50–60 years, is capable of growing gonads, completing a gametogenetic cycle and producing viable gametes [19]. Meanwhile, the new extender showed up unbalanced sex ration skewed towards females (1.6 females for each male) [19].

*Heliocidaris crassispina* is originally distributed in intertidal and shallow subtidal rocky reefs in the Pacific Ocean and the Sea of Japan in southern Japan and southeastern China [20]. It is a common herbivore in the middle part of the Sea of Japan [21]. High densities (0.8–21.6 ind./m^2^) of *H*. *crassispina* were found at a depth of 7 m in Wakasa Bay in Kyoto, Japan, where is the central distribution of *H*. *crassispina* in Japan [22]. In the western part of Wakasa Bay, the density of *H*. *crassispina* in *Sargassum* beds (0.3 ind./m^2^) is lower than that in *Corallina* beds (11.1 ind./m^2^) [23]. Along the coast of the Miura Peninsula, Kanagawa Prefecture, the dominant seaweed species are *Sargassum* spp. and *Ecklonia bicyclis* at the habitat of *H*. *crassispina*, and these seaweed species form large seaweed beds [24]. Both sex of gonads of *H*. *crassispina* are at the mature stage from May to August and the spent stage from July to August in Hirado Island, Nagasaki Prefecture, Japan [25].

In August 2014, we found the dense habitation of *H*. *crassispina*, in contrast, *Mesocentrotus nudus*, which was predominant until a couple of years ago, disappeared in shallow waters in Toga Bay along the Oga Peninsula in the Sea of Japan, Akita Prefecture in northeastern Honshu, Japan [26–27]. As the first trail to identify evidence of ad aptation occurring in response to a range extension of *H*. *crassispina* to the newly extended environments, this case study aims to explore (1) the somatic growth, and gonad size, development and color by sex of *H*. *crassispina* collected from Toga Bay, (2) the population structure (sex ratio and age composition) and subsequently to infer (3) whether the population in this range extension is likely to persist via continued recruitment from other population or self-recruitment.

## Materials and methods

### Ethics statement

*Heliocidaris crassispina* were collected from a site in the Toga Bay in Oga, Akita Prefecture, Japan that is not privately-owned or protected in any way. Field studies did not include endangered or protected species. All experimental procedures on animals were in compliance with the guidelines of Oga Fisheries Cooperative Association and Akita Prefectural Government.

### Sea urchin collection

**Fig 1:**
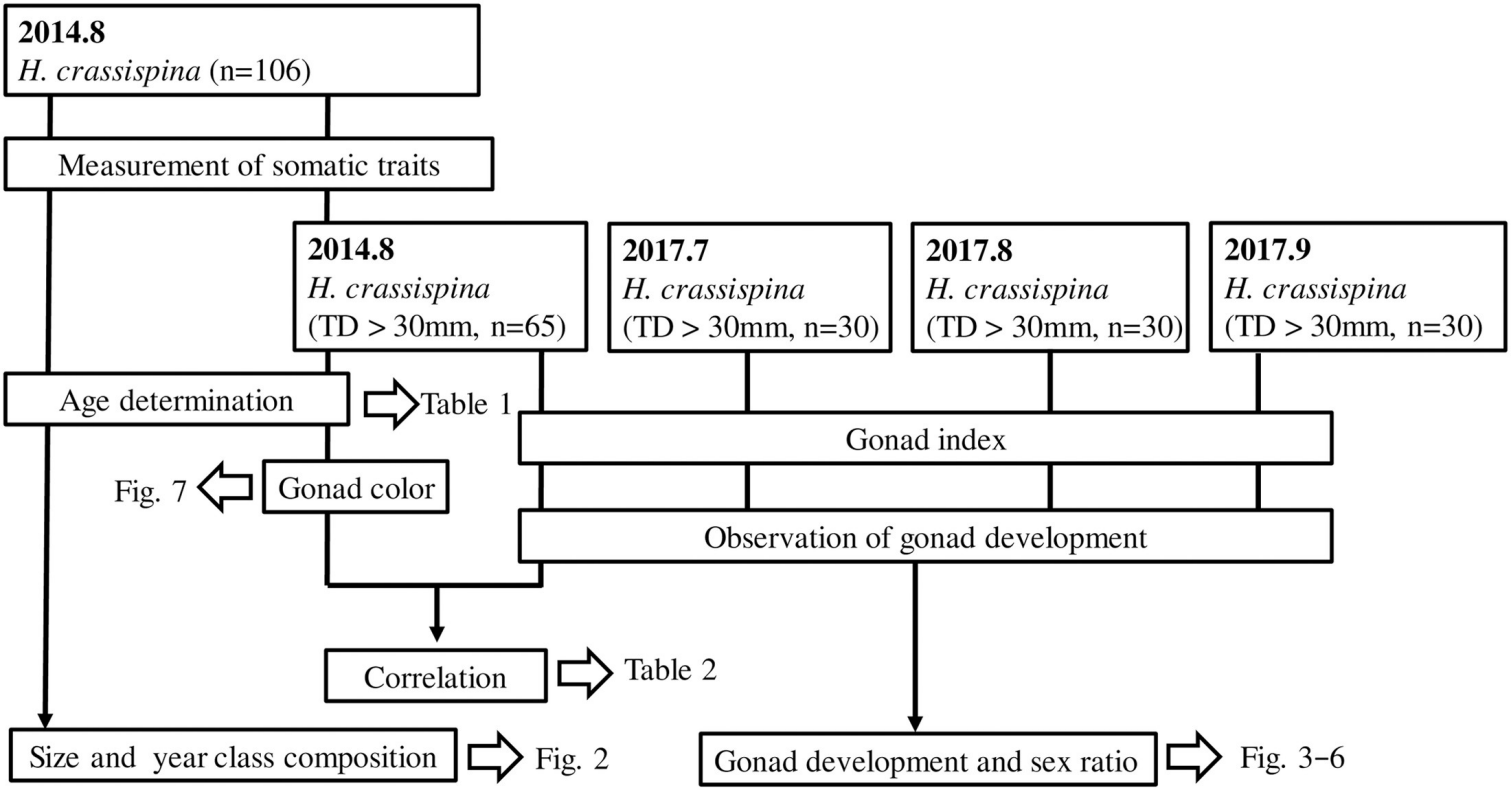
Flow diagram of the assays and the samples that were evaluated.

Fig 1 is a general flow diagram of the assays and the samples that were evaluated. A total of 106 *H*. *crassispina* were haphazardly collected in August 2014 by scuba diving in a *Sargassum siliquastrum* bed at depths of 3–5 m in Toga Bay, along the Sea of Japan coast of the Oga Peninsula, Akita Prefecture, northeastern Japan (39°57´N, 139°42´E). There, 30 *H*. *crassispina* with >30 mm diameter were collected in July, August and September 2017. Immediately after collection, the sea urchins were transported to the Marine Plant Ecology Lab, Tohoku University, Sendai, Miyagi Prefecture.

Monthly mean sea water temperature data collected over a 34-year period, from 1984 to 2017, at a depth of 2.4 m off the coast of Unosaki, Oga (39°51´N, 139°48´E), by the Akita Prefectural Institute of Fisheries, were used in this study.

### Measurement and age determination

Sea urchin test diameter (TD) (0.1 mm accuracy) was measured using a vernier caliper. Body wet weight (BW) (0.1 g accuracy) and gonad wet weight (GW) (0.01 g accuracy) were measured using an electronic balance. The gonad index (GI) (GW × 100 / BW) was calculated.

Age of 106 sea urchins collected in 2014 was determined by counting the number of black bands formed in charred genital plates [28–29], which is the age marker of this urchin species [30].

### Observation of gonad development

To observe the gonads histologically, 65 individuals with > 30 mm TD were used, because individuals with < 30 mm TD have possibly undeveloped gonads and their gonads were too small for histological observation and color measurement. One gonad (1/5 of total gonad) of each individual was preserved in 20% formalin. Using standard histological techniques, serial cross-sections (6 μm) were cut and stained with Mayer’s hematoxylin and eosin. Sections were classified based on the stage of development of the germinal cells and nutritive phagocytes (NPs): stage I, recovering; stage II, growing; stage III, premature; stage IV, mature; stage V, partly spawned; stage VI, spent [31–33]. Gonad development stages with 10 and 10–40 acini per individual female and male, respectively, were observed in the 65 urchins collected in August 2014. And 10 acini per both sex of 30 urchins were observed in July, August and September 2017. As there was a large variation in the number of acini in the testis of an individual urchin, the number of acini at each gonad developmental stage in males collected in August 2014 were converted to those per 10 acini to allow for a comparison with females. The percentage area of NPs and oocytes, ovum, and unoccupied lumen in the ovaries, and NPs, spermatocytes, spermatozoa and unoccupied lumen in testes, relative to each acinus area were measured in the same 65 urchins using imaging software (cellSence, Olympus, Tokyo, Japan). In the ovaries, the area of NPs and oocytes were combined as it was difficult to distinguish between NPs and small previtellogenic oocytes.

### Gonad color

The gonad color of the 65 sea urchins collected in August 2014, which were used for gonad development observations, were measured using a color meter (ZE-6000, Nippon Denshoku Industries Co. Ltd., Tokyo, Japan). This could detect tristimulus-values directly with flicker photometry using a 12 V, 20 W halogen lamp. L* (lightness), a* (redness), and b* (yellowness) were measured, based on the Commission Internationale de I’Eclairage color measurement system [34]. Three replicates of detection per gonad were carried out. The correlations between these color values, the gonad indices, gonad developmental stages, and ages by sex were analyzed. For analysis, gonad developmental stages I−VI were attributed as 1–6.

### Statistical analysis

The data were tested for assumptions of normality (Shapiro-Wilk test) and homogeneity of variance (Levene’s test). To stabilize the variance, the data of female body weight and total body weight at 4–7 years of age were log-transformed. Differences between the sexes in TD and BW at 4–7 years of age, GI, number of acini at each gonad developmental stage, number of each gonad developmental stage in 10 acini per urchin, and gonad color (L*, a*, b*) were analyzed by the *t*-test. Skew of sex ratio was analyzed using the chi-square test. Significant differences in TD and BW among 4–7 years of age and GI by sex among August 2014, July, August and September 2017 were analyzed by one-way ANOVA, followed by the Tukey multiple comparison test. Percentage area of gametes, NPs and oocytes, spermatocytes, NPs, and unoccupied lumen at each gonad developmental stage were analyzed by Kruskal-Wallis test followed by Steel-Dwass multiple comparison test after arcsine transformation of these data. Correlations between TD and BW and between gonad color (L*, a*, b*) and GI, gonad development, and age were estimated using Spearman’s rank correlation. Statistical analysis was done using SPSS 19.0 and JMP 10 statistical software.

## Results

### Size, year class composition, and growth

**Fig 2:**
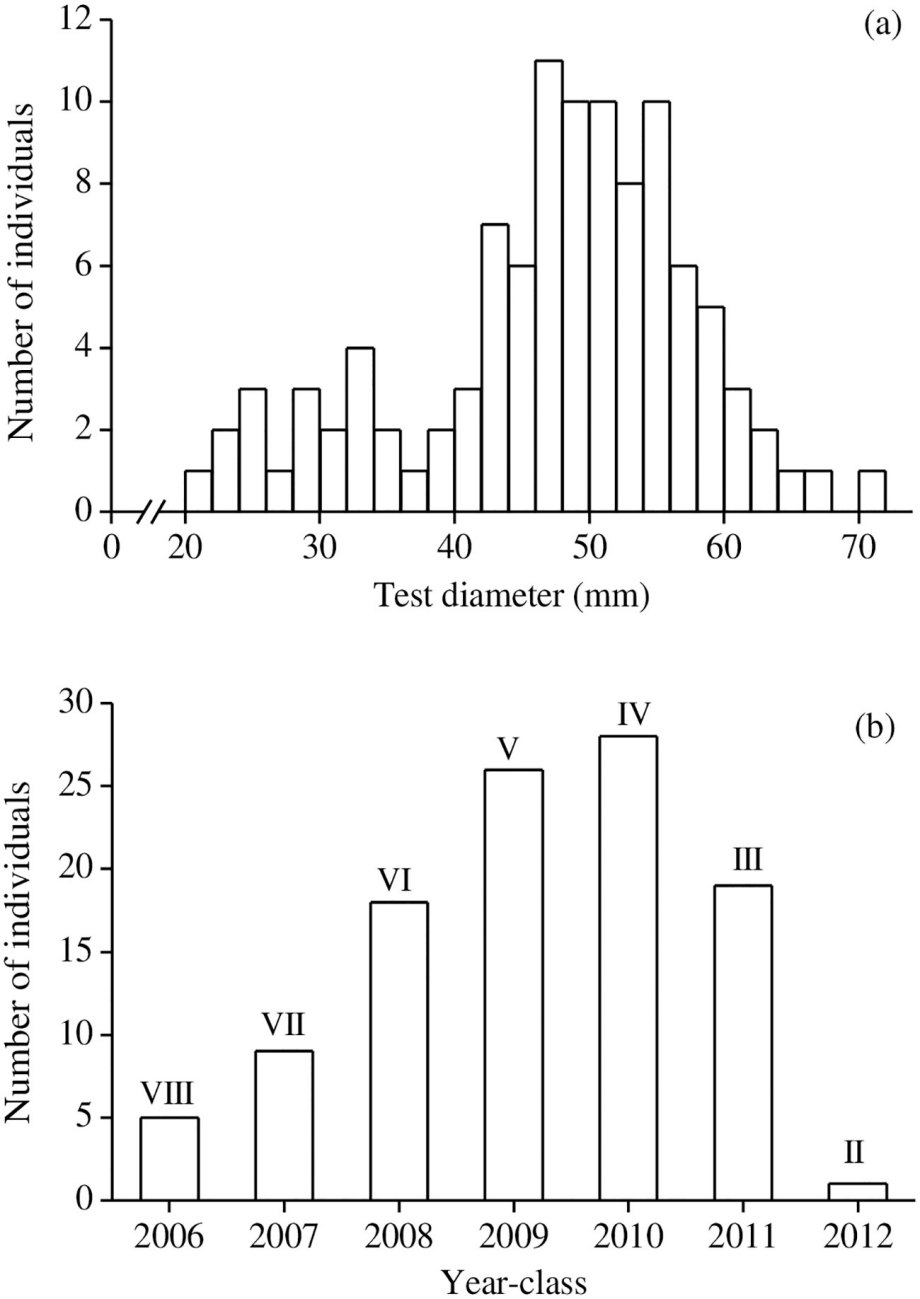
Size-frequency distribution (a) and year class composition (b) of *Heliocidaris crassispina* collected in August 2014. Roman numerals indicate ages.

TD and BW (mean ± sd) of 30 *H*. *crassispina* collected in July, August and September 2017 were 48.7 mm ± 4.5 mm and 42.7 g ± 10.9 g, 49.8 mm ± 6.1 mm and 45.4 g ± 11.1 g, and 47.0 mm ± 4.9 mm and 42.8 g ± 13.7 g, respectively. The size-frequency distribution and year class composition of 106 *H*. *crassispina* collected in August 2014 are shown in Fig 2. TD ranged from 20.4–70.9 mm. The majority of individuals occurred within the 40–60 mm TD group (Fig 2a). The sea urchins sampled consisted of seven year-classes from 2006 to 2012. There was a consistent increase in the number of urchins from the 2006 year class to the 2010 year class. In particular, the year-classes of 2010 and 2009, which are coincident with 4 and 5 years of age, respectively, were largest. (Fig 2b). TD and BW measurements for sea urchins from 3–8 years of age are shown in Table 1. There were no significant differences between male and female urchins at 4–7 years of age for either of these parameters (*p* > 0.05). For both sexes, there were significant differences in TD and BW among urchins from 4–7 years of age (*p* < 0.01). TD and BW of both sexes increased significantly between 4 and 5 years of age, with the exception of male BW. The TD of both sexes exceeded 50 mm at 5 years of age.

**Table 1:** Test diameter (TD, mm) and body weight (BW, g) (means ± SD) of female and male *Heliocidaris crassispina* (n = 65) collected in August 2014 from III−VIII years of age.

Age	♂			♀			Total		
	N	TD	BW	n	TD	BW	n	TD	BW
**III**	1	32.6	14.3	1	35	15.3	2	33.3 ± 2.4	14.8 ± 0.7
**IV**	5	46.6 ± 2.3^a^	41.2 ± 6.2^a^	10	44.7 ±3.4^a^	37.6 ± 8.3^a^	15	45.3 ± 3.1^a^	38.8 ± 7.6^a^
**V**	11	52.0 ± 2.4^b^	61.6 ± 11.4^ab^	10	51.5 ± 5.3^b^	57.7 ± 16.4^b^	21	51.8 ± 3.9^b^	59.8 ± 13.8^b^
**VI**	4	55.4 ± 2.9^bc^	70.0 ± 16.3^bc^	10	53.5 ± 6.5^b^	65.5 ± 21.8^b^	14	54.1 ± 5.6^bc^	67.7 ± 19.8^bc^
**VII**	3	58.0 ± 2.4^c^	89.1 ± 5.2^c^	6	57.7 ± 3.9^b^	78.0 ± 17.5^b^	9	57.8 ± 3.3^c^	81.7 ± 15.1^c^
**VIII**	3	64.9 ± 7.0^c^	90.7 ± 11.3^c^	1	59.3	84.7	4	62.7 ± 7.3^c^	84.8 ± 14.8^c^
**F**		16.576	12.654		9.636	11.365		18.112	20.872
**Df**		3	3		3	3		3	3
***P***		0.001	0.001		< 0.001	< 0.001		< 0.001	< 0.001

Different letters indicate significant differences between ages IV-VII (*p* < 0.05).

### Gonad development and sex ratio

There were six gonad developmental stages of the recovery (a, g), the growing (b, h), pre-mature (c, d), mature (i, j), partly spawn (e, f) and spent (k, l) in *H*. *crassispina* (S1 Fig).

**Fig 3:**
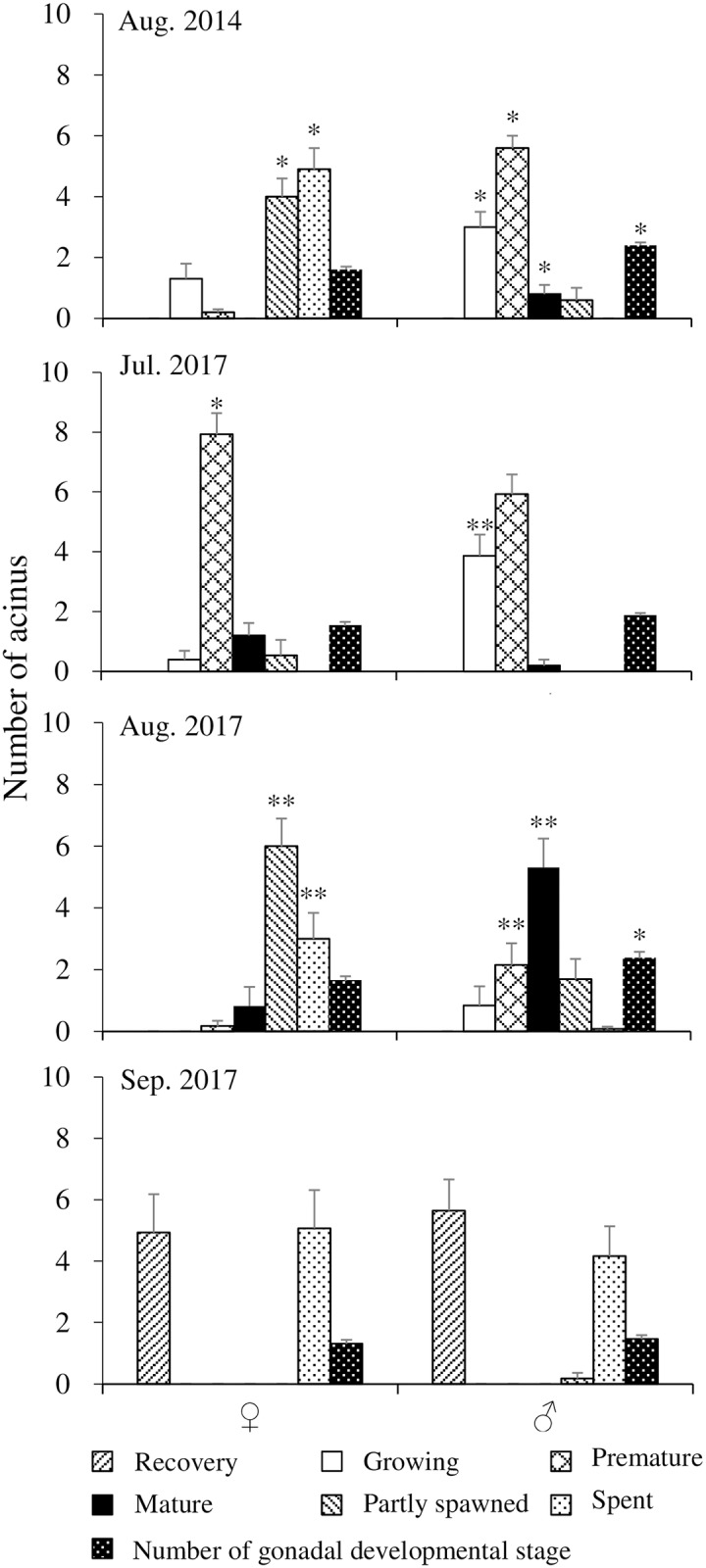
Number of acini at different gonad development stages per 10 acini and number of gonad developmental stage of *Heliocidaris crassispina* by sex in August 2014 and July–September 2017. Data indicate means ± SE. One and two asterisks indicate significant sexual differences at *p* < 0.05 and *p* < 0.01, respectively.

**Fig 4:**
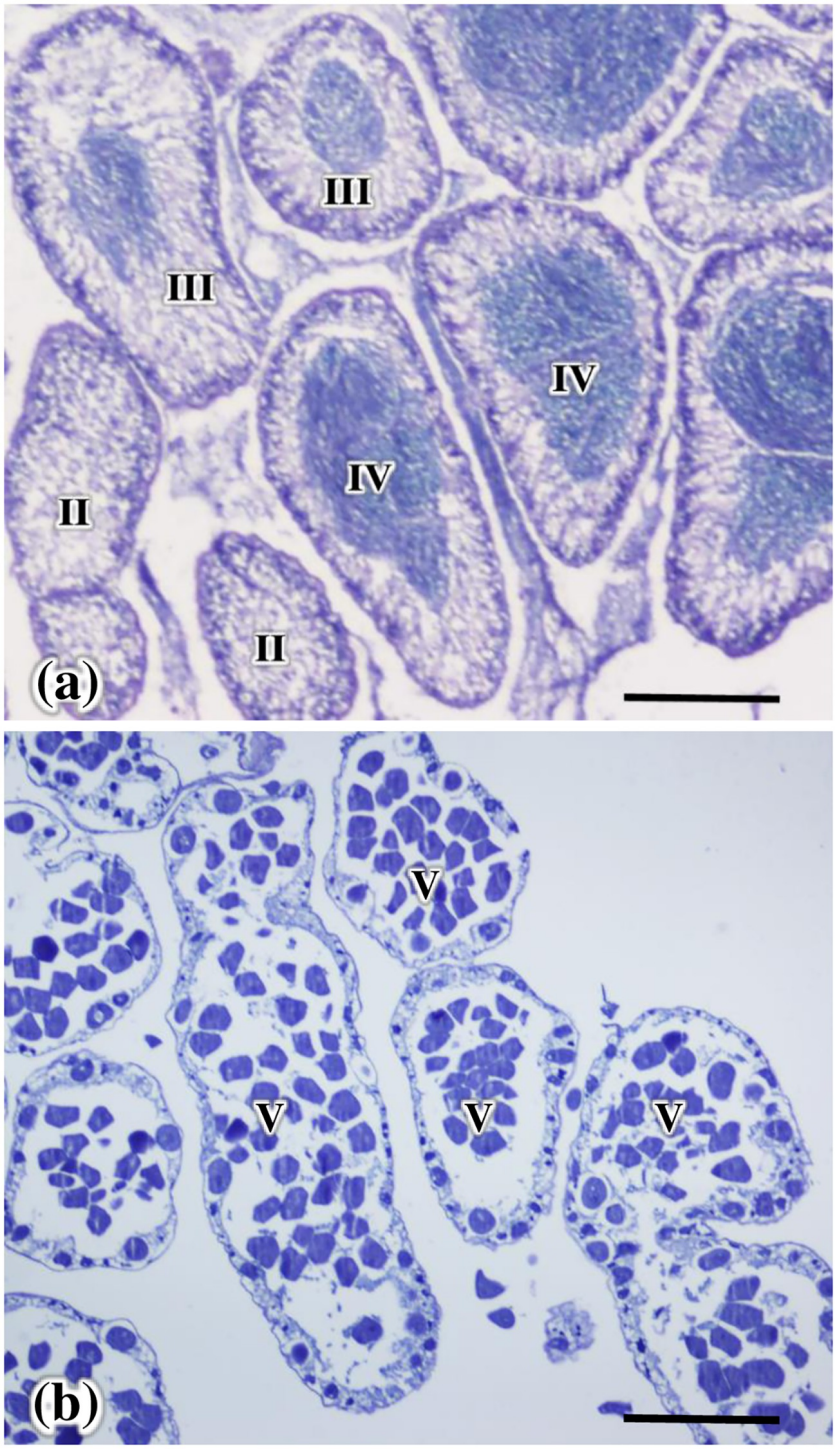
Histology of a testis (a) and ovary (b) with different development stages of acini in *Heliocidaris crassispina*. Scale bars represent 500 μm.

Number of acini at different gonad development stages, and number of gonad developmental stages in 10 acini of male and female *H*. *crassispina* are shown in Fig 3. In August 2014, the numbers of acini at the growing, premature, and mature stages in testes were significantly higher than those in ovaries. In contrast, the number at the partly spawned stage was significantly higher in the ovaries in comparison with the testes (*p* < 0.05). In July 2017, the number of acini at the growing stage in testis was significantly higher than those in ovaries, while the number at the premature stage was significantly higher than those in the testis (*p* < 0.05). In August 2017, high number of acini were seen at the partly spawned and the spent stages in females, and at premature and mature stage in males. In September 2017, high number of acini were observed at the recovery and spent stages in both sexes, and there was no significant sexual difference (*p* > 0.05). In addition, there were significantly higher numbers of gonad development stage in testicular acini than in the ovarian acini in August 2014 and August 2017 (*p* < 0.05) (Fig 3). And a testis with three different development stages of acini were simultaneously observed (Fig 4a), while only one development stage of acini was observed in the ovary (Fig 4b).

**Fig 5:**
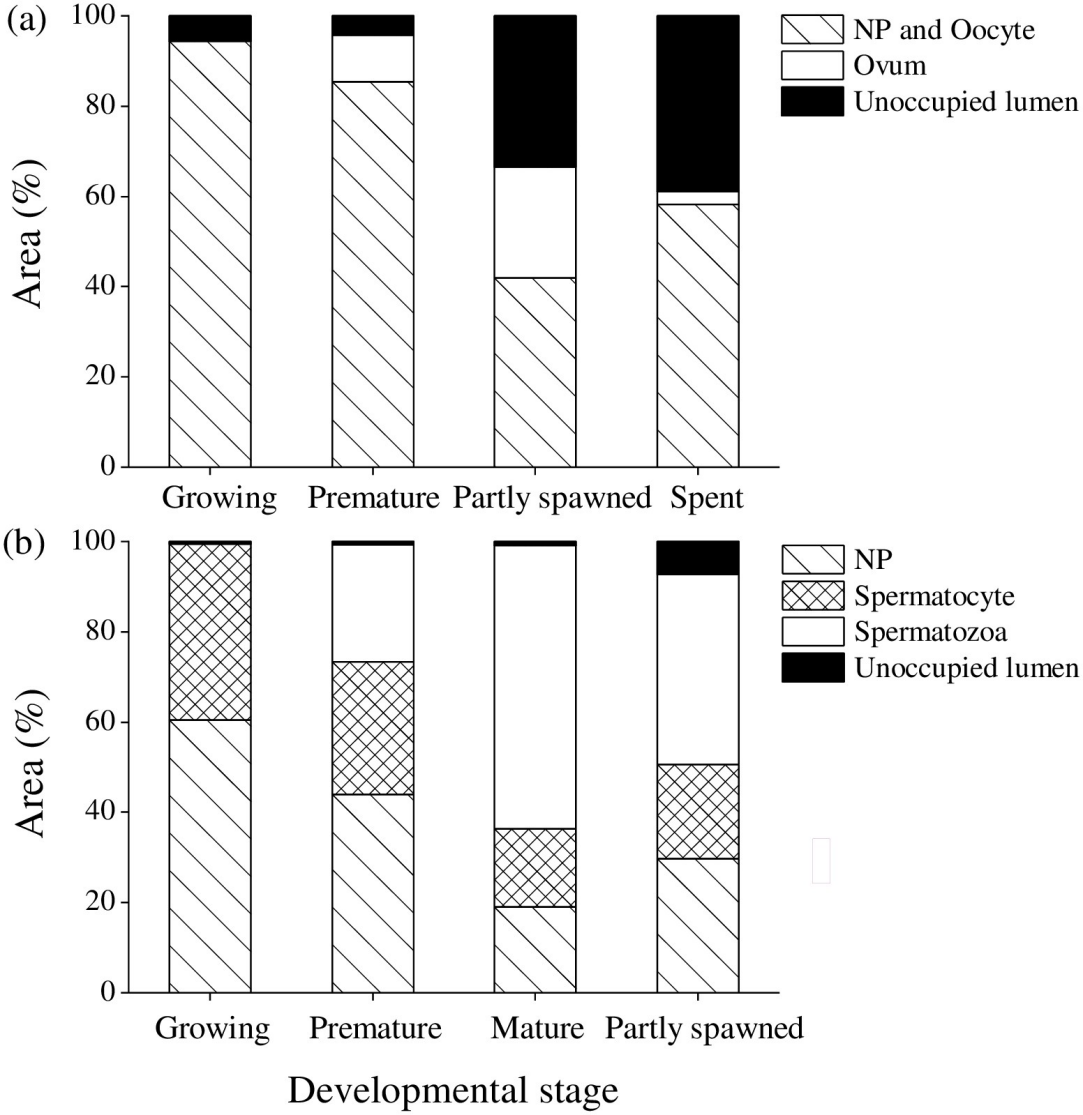
Percentage area of acinar components of female (n = 38) and male (n = 27) *Heliocidaris crassispina* at different gonad developmental stages. (a) Acinar component in ovary: nutritive phagocytes (NPs), oocytes, ovum, unoccupied lumen. (b) Acinar component in testis: NPs, spermatocytes, spermatozoa, unoccupied lumen. Urchins were collected in August 2014.

The percentage areas of acinar components of female and male *H*. *crassispina* at each gonad developmental stage are shown in Fig 5. These differed significantly among the gonad development stages in both ovarian and testicular acini (ovary: NPs and oocytes: H_3_ = 209.421, *p* = 1E-13; ovum: H_3_ = 257.706, *p* = 1E-13; lumen: H_3_ = 156.903, *p* = 1E-13; testis: NPs: H_3_ = 260.157, *p* = 1E-13; spermatocytes: H_3_ = 177.809, *p* < 0.01; spermatozoa: H_3_ = 356.254, *p* = 1E-13; lumen: H_3_ = 250.487, *p* = 1E-13). In ovarian acini, the area of ovum increased from the premature stage to its peak at the partly spawned stage, following which it significantly decreased at the spent stage (*p* < 0.01). The areas of NPs and oocytes significantly decreased from the growing stage to the partly spawned stage (*p* < 0.01). The area of lumen significantly increased from the premature stage to the spent stage (*p* < 0.01) (Fig 5a). In testicular acini, the areas of NPs and spermatocytes significantly decreased from the growing stage to the mature stage and partly spawned stage (*p* < 0.01). The area of spermatozoa significantly increased from the premature stage to the mature stage and then significantly decreased at the partly spawned stage (*p* < 0.01) (Fig 5b).

**Fig 6:**
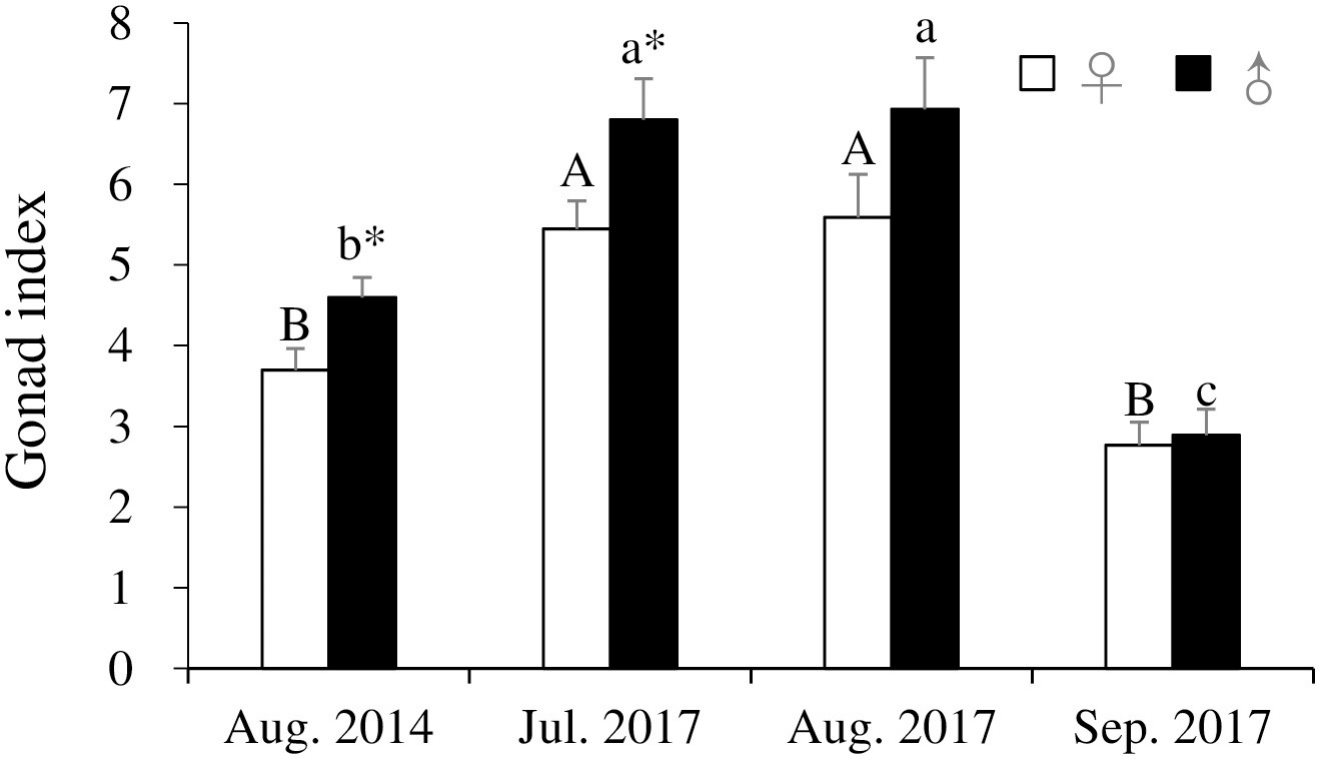
Gonad indices (mean ± SE) of *Heliocidaris crassispina* by sex in August 2014 and July–September 2017. Asterisks indicate significant sexual differences (*p* < 0.05). A and B indicate significant differences among months (*p* < 0.05).

Of the 65 sea urchins sampled, 38 were female and 27 were male, indicating a sex ratio of 1:0.7, skewed toward females, although no significant difference between sex (x^2^ = 3.723, df = 1, *p* = 0.054) was detected. GIs of *H*. *crassispina* in August 2014 and July–September 2017 are shown in Fig 6. There were significant differences of GIs in both females (df = 3, F = 11.353, *p* = 2.863E-06) and males (df = 3, F = 21.309, *p* = 7.692E-10) among months. GIs of both sexes collected in July and August 2017 were significantly higher than those in the other sampling dates (*p* < 0.05). In addition, GIs of males collected in August 2014 and July 2017 were significantly higher than those of females (*p* < 0.05).

### Gonad color

**Fig 7:**
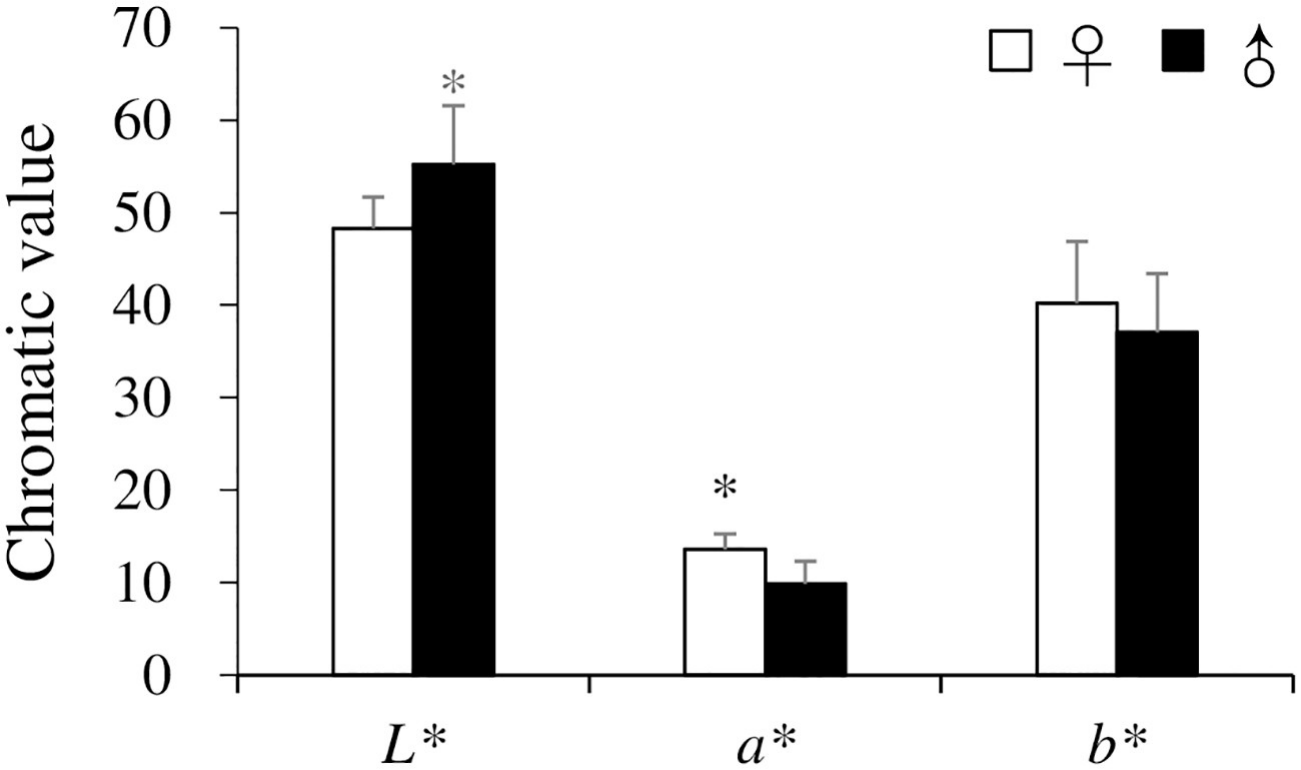
Gonad color (L*, a*, b*) (mean ± SD) of female and male *Heliocidaris crassispina* collected in August 2014. Asterisks indicate significant sexual difference (*p* < 0.05).

The testes exhibited significantly higher L* values and lower a* values in comparison with the ovaries (*p* < 0.05) (Fig 7). Spearman’s rank correlations between gonad color (L*, a*, b*), GIs, gonad developmental stages, and ages of male and female *H*. *crassispina* are shown in Table 2. A significantly positive correlation was found between L* values and GI in both sexes (*p* < 0.001). There was a significant negative correlation between a* and b* values, and gonad developmental stage and age in the females (*p* < 0.05). In males, significant negative correlations were also detected between L* values and gonad developmental stage and between b* values and age (*p* < 0.05).

**Table 2:** Spearman’s rank correlation coefficient for gonad color (L*, a*, b*), gonad index, gonad developmental stage, and age of female and male *Heliocidaris crassispina* collected in August 2014.

	Sex	N	Color	Correlation coefficient	*p*
**Gonad index**	♀	38	L*	0.511	0.001
			a*	0.160	0.337
			b*	0.255	0.123
	♂	27	L*	0.725	<0.001
			a*	−0.287	0.147
			b*	−0.002	0.994
**Gonad developmental stage**	♀	38	L*	−0.100	0.549
			a*	−0.465	0.003
			b*	−0.364	0.025
	♂	27	L*	−0.577	0.002
			a*	−0.071	0.727
			b*	0.044	0.827
**Age**	♀	38	L*	−0.262	0.113
			a*	−0.393	0.015
			b*	−0.373	0.021
	♂	27	L*	−0.003	0.987
			a*	−0.345	0.078
			b*	−0.632	<0.001

Gonad developmental stages I-VI are analyzed as 1–6.

### Seawater temperature

**Fig 8:**
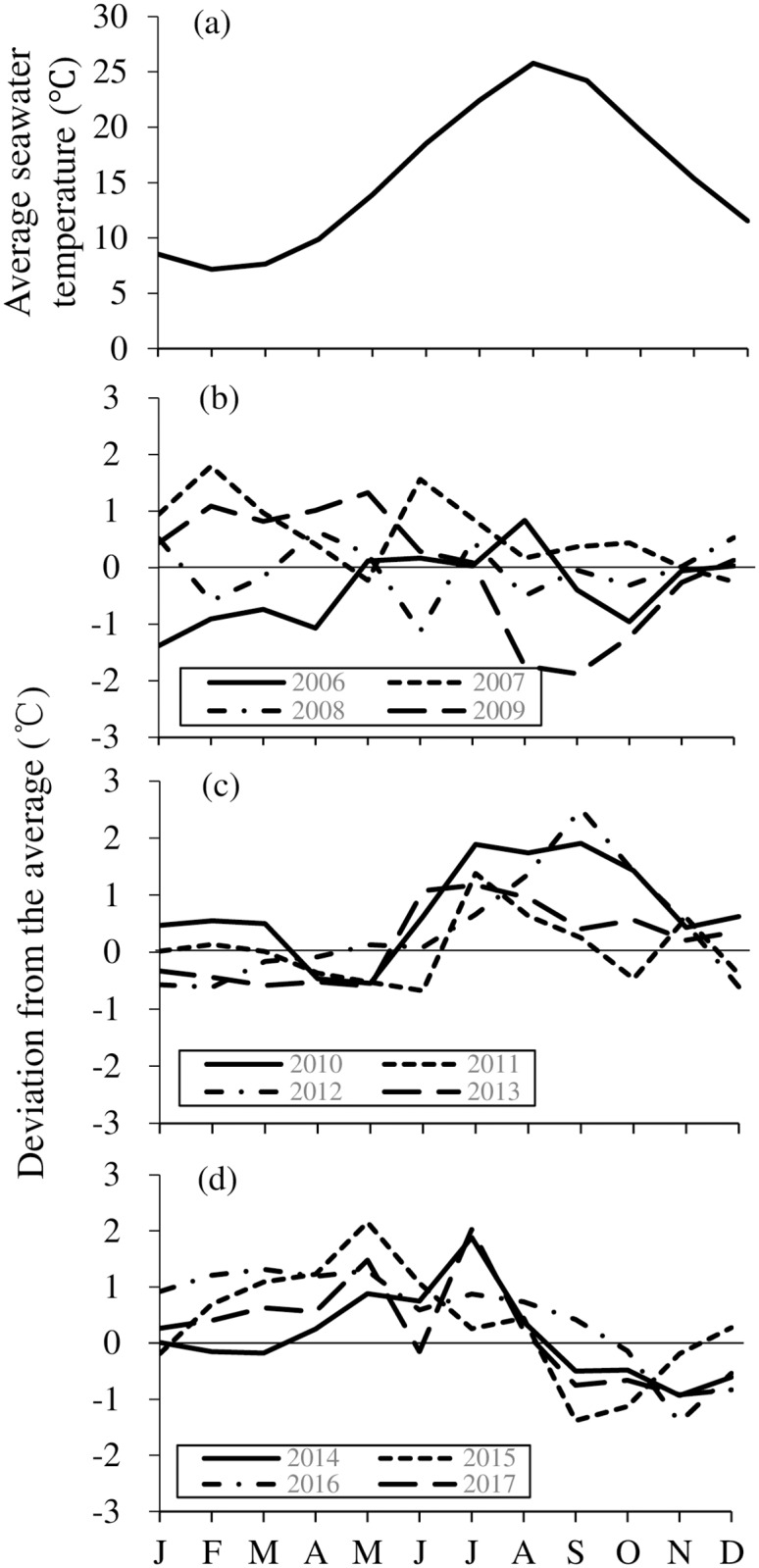
Sea surface temperature off Unosaki (39°51´N, 139°48´E), along the Oga Peninsula, Akita Prefecture. Monthly averages for 34 years (1984–2017) (a), and deviations from the average from 2006 to 2009 (b), 2010 to 2013 (c) and 2014 to 2017 (d). X-axis represents months from January (J) to December (D).

Average monthly seawater temperature for 34 years (1984–2017), and deviations from the average from 2006 to 2017, recorded at a depth of 2.4 m off Unosaki, Oga are shown in Fig 8. Average water temperature ranged from a minimum of 7.1°C in February to a maximum of 25.7°C in August (Fig 8a). The deviations in winter and spring (January–April) were higher than the average in 2007, 2009 (Fig 8b) and 2015–2017 (Fig 8d), and lower than the average in 2006, 2012 and 2013. Deviations in the summer (July–September) were higher than the average in 2010, 2012 and 2013, and lower than the average in 2009 (Fig 8b and 8c). In particular, sea surface temperatures from July to September 2010 and September 2012 were, 1.8–2.0°C (24.3–27.5°C) and 2.4°C (26.7°C) higher than the average, respectively. Higher water temperatures than the average in July and August continued during 2006–2017.

## Discussion

### Growth

Previous studies have demonstrated that sea urchin growth is directly related to the variety and availability of seaweed species present [35–37]. The growth rate of *H*. *crassispina* in a *S*. *siliquastrum* bed in Toga Bay was slow in comparison to more southern locations (S1 Table). Yatsuya and Nakahara (2004) [38] reported a high growth rate of *H*. *crassispina* reaching 46.2 mm TD at 2 years of age in *Sargassum* spp. beds. in Kodomari, in the western part of Wakasa Bay, Kyoto. The majority of the gut contents of this species were comprised of *Sargassum* spp. [23]. It has been experimentally shown that this species exhibits a preference for *S*. *serratifolium* and *S*. *thunbergii* [39–40].

Food consumption of the sea urchin species *Strongylocentrotus purpuratus*, *S*. *droebachiensis*, and *Mesocentrotus nudus* has been recorded to decrease at low water temperatures [41–43]. Verifying the food availability and the effect of low temperature on the food intake of *H*. *crassispina* along the Oga Peninsula, and associated growth rates need to be undertaken.

### Gonad development and color

Changes in the area of NPs and oocytes, ovum, and lumen from the growth to spent stages, and NPs, spermatocytes, spermatozoa, and lumen from the partly spawned stages appeared to reflect gonad development of *H*. *crassispina* occurred in the general pattern as found for other urchins [44]. The spawning season of *H*. *crassispina* is in July and August [45]. In the current study, the partly spawned and spent stages were observed in the majority of ovaries in August 2014 and 2017, which is in keeping with findings from Hirado Island, Nagasaki [25]. In contrast, delayed testicular development at the growing and premature stages in August 2014 and July 2017, and the mature stage in August 2017, in addition to higher number of different gonad developmental stages in testicular acini than those in ovarian acini were observed. These results suggested that spawning of male and female urchins was asynchronous. Optimum seawater temperature for gonad maturation in *H*. *crassispina* ranges 20–25°C [7, 46]. Low water temperature inhibited maturation of the gonad in *H*. *crassispina* [7]. Smaller number of acinus at the mature stage in testes in August 2014 than those in August 2017 may due to 0.3–0.8°C lower water temperature from February to May in 2014 than those in 2017 (7.6–15.4°C). And it is likely that spermatogenesis is inhibited by lower water temperatures within the newly extended range of this species. The GIs of male *H*. *crassispina* were higher than those of females in August 2014 and July 2017. And the average GIs in August 2014 and 2017 were 4.1 and 6.2, respectively, higher than 2.0–3.6 recorded in other localities in August and September (S1 Table). These would be due to the delayed testicular development at the premature and growth stages in August. Examination of annual cycle of GIs and gonad development in relation to water temperature would verify the sexual difference.

*Mesocentrotus nudu*s over 7 years of age exhibit brown gonad colorization in July prior to spawning. Those less than 7 years of age were found to exhibit low GIs, with values less than 5 [47]. These results roughly coincide with the findings of the current study where there was a decrease in L* values with a decreasing GI and decrease in a* or b* values with age in both sexes of *H*. *crassispina*. A reexamination of the gonad color of both sexes prior to spawning is required to allow for a comparison with southern populations.

### Population structure

Previous studies documented large spatial and temporal variations of settlement and recruitment rates of sea urchins, which were affected by abiotic and biotic factors in relation to larval supply and settlement success [48]. The occurrence of 2006–2012 year-classes of *H*. *crassispina* in the study area indicated persistent juvenile recruitment. This appears to be correlated with higher seawater temperatures than the average during the spawning period in the summer. These high temperatures persisted into the larval periods. The 2010-year class was found to have the highest abundance of individuals, which could be explained by the results of a long-term monitoring study, which indicated that typhoon disturbance increased juvenile recruitment of *H*. *crassispina* in this year, although the cause is uncertain [49]. Typhoon No. 4, which had a central pressure of 994 hPa, landed in Akita City adjacent to the Oga Peninsula in August 2010 [50] may have resulted in increased juvenile recruitment in Oga.

The sex ratio was found to be skewed toward females, although this was not found to be statistically significant. This was in keeping with research carried out on *C*. *rodgersii* with an extended range in northern New Zealand [19]. This suggested that the newly extended population established also had an unbalanced sex ratio [51–52]. A low percentage of males in the population would limits sperm concentration during spawning and decreases the success rate of fertilization in marine invertebrates [53].

### Reproduction

Range extensions in the distribution and abundance of marine organisms are directly driven by temperature [5,13,19,54–57]. The average annual sea surface temperature in the central Sea of Japan has increased since 1999, with values that were higher than average recorded until 2014 [50]. In August 2010 and 2012, extremely high sea surface temperatures of 27.5°C and 27.1°C, respectively, were recorded in Oga. Tsuji et al. (1989, 1994) [58–59] reported that *H*. *crassispina* and *M*. *nudus* were distributed at depths of < 4 m and > 4 m, respectively, on the west coast of Wakasa Bay, Kyoto, where high seawater temperatures over 28°C in the summer of 1994 caused a mass mortality of *M*. *nudus*. As a result of this mass mortality, *H*. *crassispina* extended into the deeper regions previously inhabited by *M*. *nudus* [22]. Based on the age composition data, *H*. *crassispina* extended its range into Toga Bay in 2006, when the summer (August) sea surface temperature was high. These high sea surface temperatures in July and August continued until 2017. Therefore, it is most likely that the range extension of *H*. *crassispina* from historic habitat was due to an increase in the sea surface temperatures, particularly in the summer.

Ling et al. (2008) [56] reported that the poor development of *C*. *rodgersii* larvae at temperatures below 12°C indicated that this species had not undergone an adaptive shift to the cooler Tasmanian environment. In contrast, in northern New Zealand, the lower thermal threshold of the larvae was 16°C [19]. In Tasmania, there was a strong pattern of decreasing sea urchin age and abundance with increasing distance from the historic range, being consistent with a model of range extension driven by a recent change in patterns of larval dispersal [5]. In the current study, delayed testicular development suggest that the newly extended population maintain by recruits of larvae transported from southern population. Further research is required to determine the lower thermal threshold of *H*. *crassispina* larvae to determine whether self-recruitment is possible in the extended population. In addition, it should be determined whether an Allee effect is present in the extended population [59–60], which may decrease growth rate [61].

The *H*. *crassispina* which have extended their range to the Oga Peninsula appeared to have a slower growth rate in comparison with southern populations. They also exhibited a delay in testicular development, but not ovarian development. A retention of testicular acini with greatly varied gonad developmental stages was first observed in sea urchin gonads. This indicated that the spawning of both sexes was asynchronous. It is likely that the range extension of *H*. *crassispina* was due to an increase in water temperatures, particularly summer temperatures. It has not yet been established whether the newly extended population established by self-recruitment and/or larval transport from southern populations. Further research is required to determine the lower thermal threshold of *H*. *crassispina* larvae and to compare the abundance of the extended population with southern populations.

## Supporting information

S1 Fig Histology of testis (a-f) and ovaries (g-l) of *Heliocidaris crassispina*. a and g: recovery stage; b and h: growth stage; c and i: premature stage; d and j: mature stage; e and k: partly spawned stage; f and l: spent stage; NP: nutritive phagocyte, PO: previtellogenic oocyte; VO: early vitellogenic oocyte; SC: spermatocyte; O: ovum; S: spermatozoa; L: lumen; R: residual ovum. Scale bars represent 100 μm in all images. Description: In the gonads, the recovering stage with small numbers of primary spermatocytes or previtellogenic oocytes along the acinal wall and with NPs filling the lumen (S1a and S1g Fig), the growing stage with increasing numbers of spermatocytes or early vitellogenic oocytes along the acinar wall and with NPs filling the lumen (b, h), the premature stage with spermatozoa or ova at the center of the lumen, and with spermatocytes or vitellogenic oocytes along the acinar wall (c, i), the mature stage with spermatozoa or ova filling the lumen, and with spermatocytes or vitellogenic oocytes along the acinar wall (d, j), the partly spawned stage with spermatozoa or ova less concentrated and with spaces in the lumen (e, k) and the spent stage with some relict spermatozoa or ova and empty spaces in the lumen (f, l).
(PDF)

S1 Table Test diameter (TD) at ages I-V, and gonad indices during spawning seasons at different seaweed beds. n.d.: no description.
(PDF)

S1 File Raw data. All data was separated in different sheets, according to the sampling time.
(XLSX)
