# Supplementary figures and images for: Reproduction and population structure of the sea urchin Heliocidaris crassispina in its newly extended range: The Oga Peninsula in the Sea of Japan, northeastern Japan
Source: PLoS One. 2019 Jan 2;14(1):e0209858. doi: 10.1371/journal.pone.0209858 (PMC6314614; doi:10.1371/journal.pone.0209858)

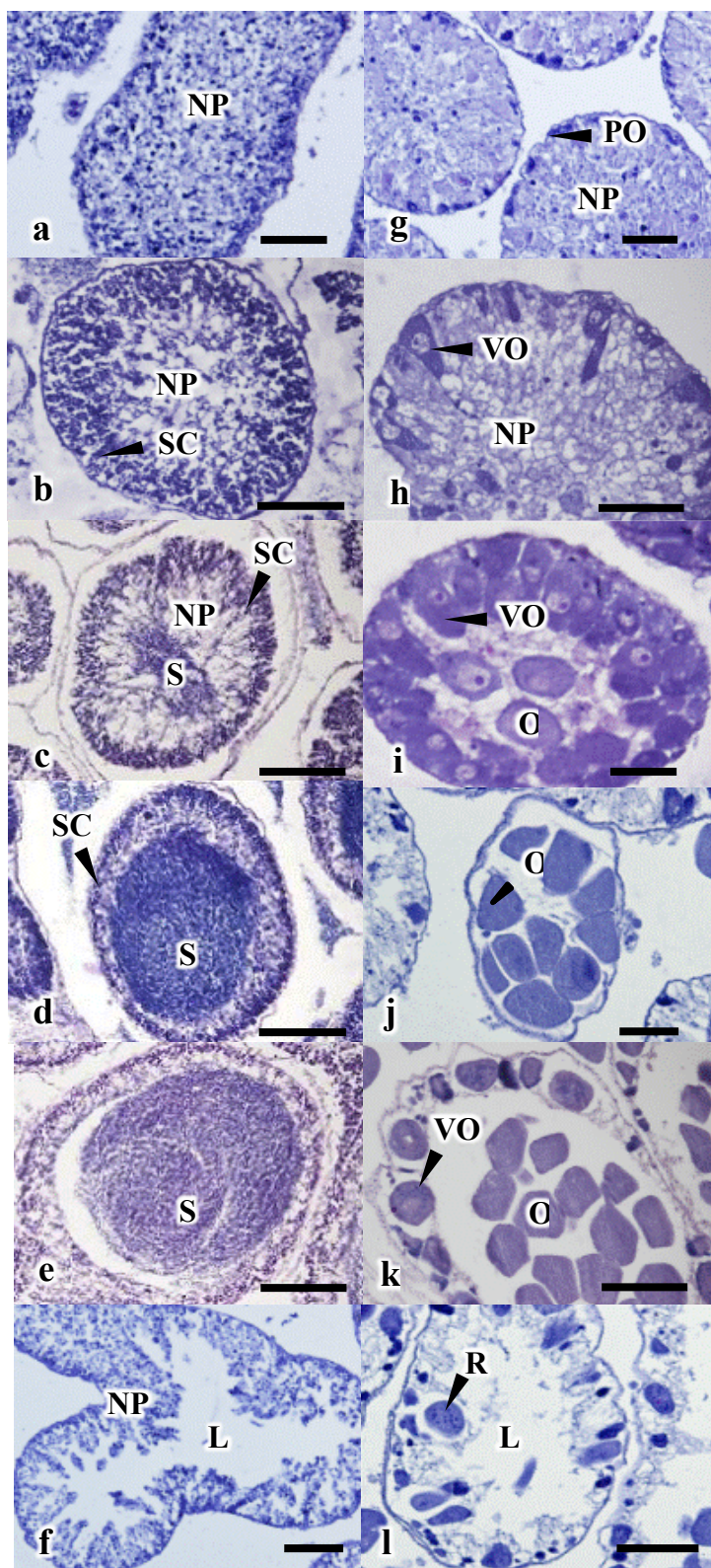

Supplement: S1 Fig — a and g: recovery stage; b and h: growth stage; c and i: premature stage; d and j: mature stage; e and k: partly spawned stage; f and l: spent stage; NP: nutritive phagocyte, PO: previtellogenic oocyte; VO: early vitellogenic oocyte; SC: spermatocyte; O: ovum; S: spermatozoa; L: lumen; R: residual ovum. Scale bars represent 100 μm in all images. Description: In the gonads, the recovering stage with small numbers of primary spermatocytes or previtellogenic oocytes along the acinal wall and with NPs filling the lumen (S1a and S1g Fig), the growing stage with increasing numbers of spermatocytes or early vitellogenic oocytes along the acinar wall and with NPs filling the lumen (b, h), the premature stage with spermatozoa or ova at the center of the lumen, and with spermatocytes or vitellogenic oocytes along the acinar wall (c, i), the mature stage with spermatozoa or ova filling the lumen, and with spermatocytes or vitellogenic oocytes along the acinar wall (d, j), the partly spawned stage with spermatozoa or ova less concentrated and with spaces in the lumen (e, k) and the spent stage with some relict spermatozoa or ova and empty spaces in the lumen (f, l). (PDF) [file pone.0209858.s001.pdf]
