# Supplementary material for: Reproduction and population structure of the sea urchin Heliocidaris crassispina in its newly extended range: The Oga Peninsula in the Sea of Japan, northeastern Japan
Source: PLoS One. 2019 Jan 2;14(1):e0209858. doi: 10.1371/journal.pone.0209858 (PMC6314614; doi:10.1371/journal.pone.0209858)
Supplement: S1 Table — n.d.: no description. (PDF) [file pone.0209858.s002.pdf]

Habitat	Dominant seaweed	TD (mm)					Gonad index		Reference
		I	II	III	IV	V			
**Toga Bay, Akita**	*Sargassum siliquastrum*	7.2	20.9	31.9	41.6	48.8	Aug.	4.1	This study
**Kodomari, Kyoto**	*Sargassum* spp.		46.2				Sep.	3.6	[38]
	Articulated Corallines		33				Aug.	3.4	
**Miura, Kanagawa**	*Colpomenia sinuosa*	18.1	32.9	43.8	51.8	57.7	Aug.	4.1	[24]
	Ecklonia bicyclis								
	Sargassum spp.								
**Hongkong, China**	n.d.	14.3-19.5	26.9-33.3	37.0-44.2	45.2-52.5	52.6-57.6			[30]
**Hirado, Nagasaki**	n.d.						Aug.	≈3	[25]
**Mera, Shizuoka**	n.d.						Aug.	2-3	[40]
